# Numerical Simulation of Electromagnetic–Thermal–Fluid Coupling for the Deformation Behavior of Titanium–Aluminum Alloy under Electromagnetic Levitation

**DOI:** 10.3390/ma17133338

**Published:** 2024-07-05

**Authors:** Xiaoliang Wang, Guifang Zhang, Peng Yan, Xinchen Pang, Zhixiang Xiao

**Affiliations:** 1 Faculty of Metallurgical and Energy Engineering, Kunming University of Science and Technology, Kunming 650093, China; wangxiaoliang@kust.edu.cn (X.W.); xinchen001118@163.com (X.P.); xiaozhixiang1998@163.com (Z.X.); 2 Key Laboratory of Clean Metallurgy for Complex Iron Resources in Colleges and Universities of Yunnan Province, Kunming University of Science and Technology, Kunming 650093, China

**Keywords:** EML, Ti–Al alloy, ALE, electromagnetic–thermal–fluid field coupling, oscillatory deformation

## Abstract

Electromagnetic levitation (EML) is a good method for high-temperature processing of reactive materials such as titanium–aluminum (Ti–Al) alloys. In this study, the oscillation and deformation processes of Ti-48Al-2Cr alloy specimens at different high-frequency currents during the EML process were simulated using the Finite Element Method and Arbitrary Lagrangian–Eulerian (ALE) methods. The data of oscillation, stabilization time, deformation, and distribution of electromagnetic–thermal–fluid fields were finally obtained. The accuracy of the simulation results was verified by EML experiments. The results show the following: the strength and distribution of the induced magnetic field inside the molten droplet are determined by the high-frequency current; under the coupling effect of the electromagnetic field, thermal field, and fluid field, the temperature rise of electromagnetic heating is rapid, and accompanied by strong stirring, resulting in a uniform distribution of the internal temperature and a small temperature difference. Under the joint action of gravity and Lorentz force, the molten droplets are first within a damped oscillation and then tend to stabilize with time, and finally maintain the “near rhombus” shape.

## 1. Introduction

Titanium–aluminum (Ti–Al) alloys are widely used in aerospace applications as a high-temperature structural material due to their low density, high specific strength, and good flame-retardant properties [1,2,3,4]. However, despite these advantages, their low ductility and low-temperature fracture toughness limit their application [5]. Electromagnetic levitation (EML) technology is a type of non-contact melting technology that has many advantages, such as non-contact, fast heating speed, wide temperature range, and internal uniformity, among others [6,7,8,9,10]. By subjecting metal alloy materials to deep subcooling and rapid cooling treatment, EML can significantly alter the morphology and composition of the solidification organization of metal alloys, thereby improving the overall performance of alloy materials [11,12,13,14]. EML offers several advantages in the production of high-performance Ti–Al alloys, including significantly reducing material contamination to maintain purity, promoting composition homogenization, allowing finer control of the cooling process, thereby tuning the microstructure, reducing thermal stresses and microcracking, improving mechanical properties, and increasing energy efficiency and precision of heating control. Due to the levitation and oscillation behavior of liquid metals during EML is complex and affects alloy preparation, conventional experiments are unable to investigate the role of magnetic, thermal, and fluid fields inside the molten droplets. Many researchers have investigated the deformation of molten droplets during EML by simulation methods, taking into account the mutual coupling of electromagnetic, thermal, and fluid fields.

Kermanpur et al. [15] developed a simulation model that coupled electromagnetic and thermal fields. They verified it using an analytical solution and experimental results. Asakuma et al. [16] also performed a numerical simulation of the deformation behavior of silicon droplets under EML. Their simulation was based on a mathematical model that used mixed finite elements and boundary elements. The simulation results showed that the droplets gradually became flat with an increase in current. Liang et al. [17] studied the deformation mode and flow behavior of liquid Ti–Al–Nb alloys at high temperatures, over a wide current range of 700–1400 A, using numerical simulations based on the arbitrary Lagrangian–Eulerian method, along with corresponding EML experiments. The arbitrary Lagrangian–Eulerian (ALE) method combines the advantages of the Eulerian and Lagrangian methods and has been widely used to study fluid dynamics problems, such as those involving free surfaces, large deformations, and fluid–solid coupling [18,19]. To effectively control the solidification organization of Ti–Al-based alloys, it is necessary to study the effect of currents on the levitation behavior and internal transfer behavior of these alloys.

In this study, we have established a mathematical model using a combination of FEM and ALE methods to simulate and predict the behavior of Ti-48Al-2Cr alloy droplets at different currents. This model considers the coupling of the electromagnetic, thermal, and fluid fields, and analyzes the oscillation, deformation, and transfer of the internal electromagnetic, thermal, and fluid fields. The results provide valuable insights for controlling the levitation stability of titanium–aluminum alloys during the EML melting process and regulating the internal organization of materials.

## 2. Physical and Numerical Models

### 2.1. Physical Model

**Figure 1:**
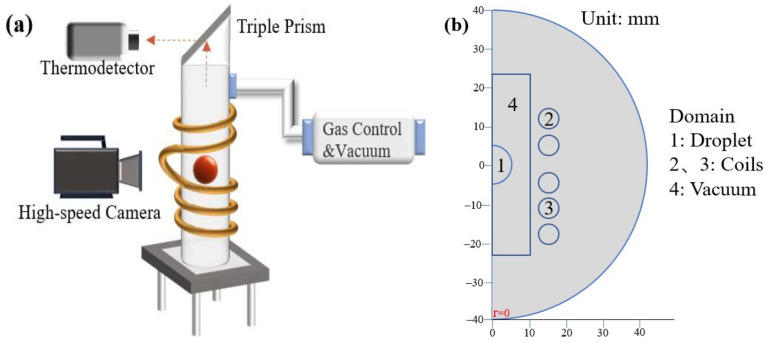
EML system: (**a**) EML schematic; (**b**) EML simulation computation domain.

The EML system used in the experiment mainly includes a high-frequency power supply, pure copper coil, quartz tube, triangular prism, pure copper crucible, levitation specimen, gas inlet and outlet, thermodetector and high-speed camera, and so on. The Ti-48Al-2Cr alloy used in the EML experiment was obtained by melting high-purity Ti grains (Ti, 99.99 wt%), high-purity Al grains (Al, 99.9 wt%) and high-purity Cr grains (Cr, 99.95 wt%) in a vacuum-water-cooled copper crucible arc furnace after repeated melting times to ensure the homogeneity of the compositions, and the mass was 1.2 g. The schematic of the EML is shown in Figure 1a. The specimen is placed in a vacuum environment, where a high-frequency harmonic magnetic field is then applied to it. This causes electromagnetic induction, which generates eddy currents. The eddy currents are concentrated on the surface of the specimen due to the skin effect. The coil consists of two sections, namely the upper and lower parts, where the upper part of the coil is two turns, and the lower part of the coil is three turns.

(1) In the lower part, a specimen with a moderate mass made of a Ti–Al alloy is levitated by the Lorentz force. It is then quickly heated up and melted due to Joule heat.
(2) The upper part is designed to prevent oscillation of the specimen.

The stabilizing coil is connected in series with the levitation coil but in the opposite direction. Figure 1b shows the simulation domain, where domain 1 is the Ti–Al alloy specimen, domains 2 and 3 are the coils, and domain 4 is vacuum domain.

### 2.2. Mathematical Model

In this study, the transient behavior of the alloy in the frequency domain was simulated using COMSOL 5.6 multiphysics field software, which considers three modes of physical field coupling, namely the electromagnetic, fluid, and thermal fields, according to the characteristics of electromagnetic levitation [17,20]. At each time step, the Joule heat and Lorentz force generated by the harmonic electromagnetic field are transferred to the thermal and flow fields, respectively, and the Lorentz force, gravity, and surface tension are applied to the alloy molten droplets to predict the oscillations, deformations, and flow fields of the melt. Dynamic meshing (ALE method) was used throughout the calculations, and the results were re-meshed at each time step to analyze the exact shape of the melt drop and the oscillation process. When the set time is reached, the simulation ends and the results for the different physical fields are released. The multiphysics field coupling considered by the calculation is specifically where the Lorentz force couples the electromagnetic field to the fluid field, while the non-isothermal flow couples the fluid field to the thermal field, simplifying the Navier–Stokes equations with the Boussinesq approximation. This multiphysics field coupling method considers the bidirectional influence of different fields and provides higher computational accuracy compared to traditional methods.

#### 2.2.1. Assumptions

Based on the related literature [16], this study makes some assumptions about the model:

(1) Since the numerical model is a two-dimensional axisymmetric model, only the oscillations occurring in the vertical direction are considered;
(2) In the fluid model, both the molten droplet and the gas are set as incompressible fluids, and the pressure generated by the gas flow on the surface of the suspended droplet is ignored;
(3) The mass of the molten droplet is constant without considering the volatilization that occurs when the temperature of the droplet is above the liquid phase line.

#### 2.2.2. Mathematical Equations

The electromagnetic field generated during EML melting is described by a system of Maxwell’s equations as follows [21]:

(1) ∇×H=J+∂D/∂t

(2) ∇×E=−∂B/∂t

(3) ∇⋅B=0

(4) ∇⋅D=ρ

where ***H*** is the magnetic field intensity, ***E*** is the electric field intensity, ***B*** is the magnetic induction intensity, ***D*** is the electric flux density, ***J*** is the current density, and *ρ* is the electric density.

In order to solve the Maxwell equations, a magnetic vector potential ***A***, and an electric scalar potential *V*, are defined as follows:

(5) B=∇×A

(6) $$E=-\frac{\partial A}{\partial t}-\nabla V$$

The Lorentz Force and Joule heat generated would be used to analyze the fluid and thermal fields, respectively. The time-averaged Lorentz force ***F*** and Joule heat *Q* inside the specimen can be calculated using the following equations:

(7) F=12Re(J×B∗)

(8) Q=12Re(J⋅J∗σ−1)

where *σ* is the conductivity, Re represents the real part of a complex quantity, and the asterisk designates the complex conjugate.

The governing equations for the thermal and flow fields (incompressible and Newtonian fluids) for the transient analysis are as follows:

(9) ρ∇⋅u=0

(10) $$\rho \partial u/\partial t+\rho u\cdot (\nabla u)=\nabla \cdot \left\{-pI+\mu \left[\nabla u+{\left(\nabla u\right)}^{T}\right]\right\}+F+\rho g_{\mathrm{const}}+F_{st}$$

(11) $$\rho C_{p}\left[\partial T/\partial t+u\cdot \nabla T\right]+\nabla \cdot (-k\nabla T)=Q-\nabla \cdot q_{s}$$

where *ρ* is the density of the alloy, *μ* is the viscosity, which varies with temperature, *k* is the thermal conductivity (which varies with temperature), ***u*** is the velocity vector, ***I*** is the unit-diagonal matrix, $F_{st}$ is the surface tension, *T* is the temperature,p is the pressure, *C_p_* is the corrected specific heat taking into account the latent heat, and $q_{s}$ is the surface to ambient radiation heat.

The surface tension $F_{st}$ is defined by the following:

(12) $$F_{st}=\nabla \cdot [\gamma (I-{\mathrm{nn}}^{T})\delta ]$$

where *γ* is the surface tension coefficient, ***n*** is the interface unit normal, and *δ* is a Dirac delta function, nonzero only at the fluid interface.

The Lorentz force ***F*** in the vertical direction is responsible for the buoyancy force ***F*_z_** acting on the droplet. The kinetic equation governing the vertical oscillatory motion of the droplet is given as follows:

(13) $$d(u,t)=(F_{z}-\frac{4}{3}\pi r^{3}\rho \cdot g_{const})/(\frac{4}{3}\pi r^{3}\rho )$$

#### 2.2.3. Initial and Boundary Conditions

The titanium–aluminum alloy specimen was initially in a sphere, levitated in a high-frequency harmonic magnetic field between two sets of coils. The weight of the Ti–Al sample was 1.2 g, and its radius was calculated to be about 4.17 mm based on its density, which was placed in a vacuum environment. The initial levitation position was 0 mm away from the center of the coils. The initial and boundary conditions for different physical fields are provided below.

(1) The boundary condition for fluid flow at the droplet surface is given by the following:
  (14) $$(-pI+\mu (\nabla u+\nabla u^{T}))\cdot n=\gamma \xi \cdot n$$
  where *ξ* is the local average curvature of the surface.
(2) Radiation of heat from the surface to the environment is as follows:
  (15) $$-n\cdot q_{s}=\varepsilon_{r}\sigma_{r}(T_{amb}^{4}-T^{4})$$
  where *T* is the sample temperature, *T*_amb_ is the ambient temperature, and *ε*_r_ and *σ*_r_ are the emissivity and Boltzmann constant, respectively.
(3) Initial conditions for the thermal and flow fields include the following: *T*_int_ = *T*_amb_ = 298 K, *u* = 0, *p*_0_ = 101 kPa.

### 2.3. Model Parameters

The main physical parameters of the numerical simulation are shown in Table 1. The coil material is copper with a conductivity of 5.99 × 10^7^ S/m; the power supply type is alternating current with a current range of 462 A to 546 A and a frequency of 327 kHz. Since the EML process is to control the power of the high-frequency power supply to realize the stable levitation of the titanium–aluminum alloy specimen, we set five groups of levitation power in the experiment and read the corresponding currents accordingly, which were 462 A, 476 A, 504 A, 532 A, and 546 A. Therefore, these five values of currents were selected for the numerical simulation.

## 3. Results and Discussion

### 3.1. Simulation of Electromagnetic Levitation Multiphysics Field

#### 3.1.1. Simulation Validation

**Figure 2:**
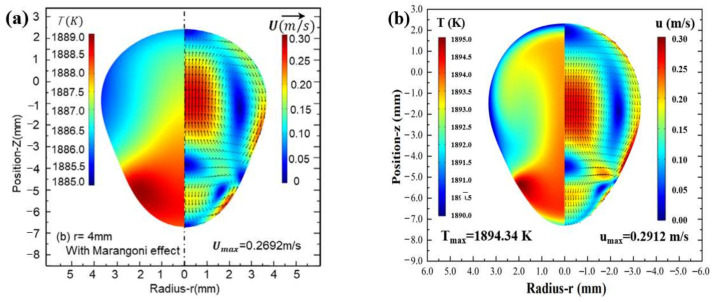
Numerical simulation verification results of temperature and fluid field of silicon droplet: (**a**) relevant literature [24]; (**b**) this study.

In order to assess the reliability of the model used in our study, the same parameters reported in the literature [24] were used in the numerical model, with the same dimensions of the coil structure and identical parameters of the molten droplets, and the same were all considered for the coupling of the electromagnetic field, the flow field, and the temperature field, which was finally verified by the magnitude and distribution of the temperature field. Figure 2 shows the states of the droplets after stabilization of levitation. Figure 2a shows the results of the temperature and fluid fields of the silicon molten droplet in the comparative literature, and Figure 2b shows the results obtained by the numerical model established in this study. It can be seen that the maximum temperature in the molten droplet calculated in this model was 1894.34 K and the maximum flow rate was 0.2912 m/s; the maximum temperature in the comparative literature was 1889 K and the maximum flow rate was 0.2692 m/s. The deviation in temperature was 0.26%, while the deviation in flow rate was 8.2%, which is less than the 10% error margin. The distribution of the thermal field and the fluid field derived from the simulation are essentially the same, indicating that the numerical model in this study is reliable.

#### 3.1.2. Simulation of Electromagnetic Field

**Figure 3:**
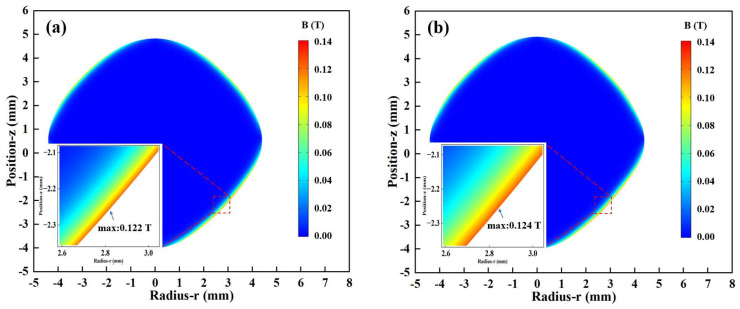

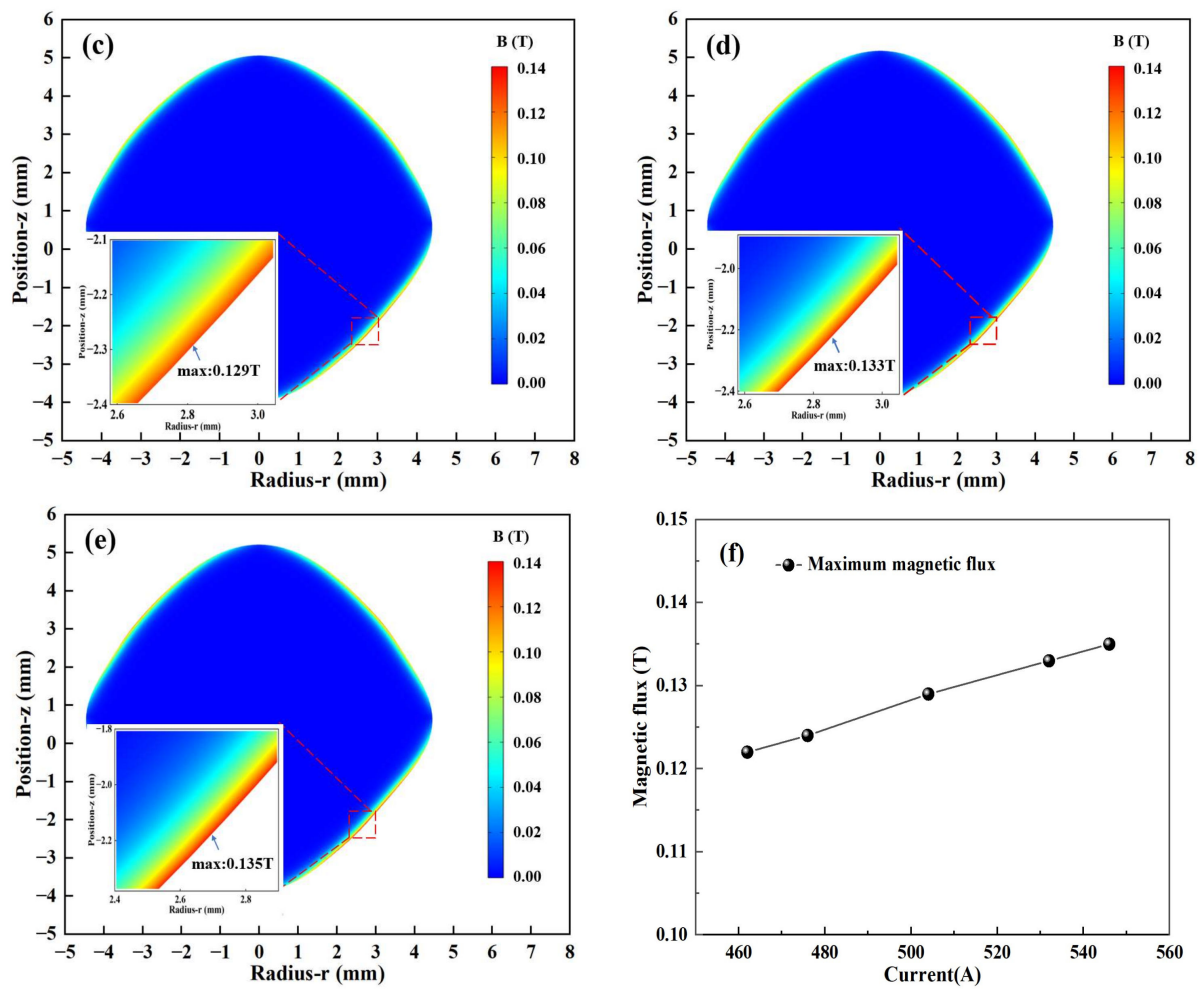
Simulation results of the magnetic field at different currents: (**a**) 462 A; (**b**) 476 A; (**c**) 504 A; (**d**) 532 A; (**e**) 546 A; (**f**) Maximum magnetic flux density.

In this study, the frequency domain transient is selected, and the simulation calculation time is taken as 5 s, considering the stabilization time of the molten droplet. The distribution of magnetic flux density inside the molten droplet at different currents, calculated from the Equations (1)–(6), is shown in Figure 3. The magnitude of the high-frequency current determines the strength of the induced magnetic field inside the droplet. Consistent with the skin effect, the high-frequency current in the levitation coil also determines the distribution of the induced magnetic field inside the molten drop in the form of a thin wall near the surface, as shown in Figure 3a–e. Since the magnetic flux density is also proportional to the turns of coils, in the longitudinal direction, the distribution in the upper half of the droplet is weaker than that of the lower half, and the strongest regions are located in the lower left and lower right, and the maxima increase almost proportionally with the increase in the current, 0. 122 T, 0.124 T, 0.129 T, 0.133 T, and 0.135 T. The weakest induced magnetic field is found in the central region of the droplet, which is small and negligible. In addition, the distribution of the induced magnetic field generated on the surface of the droplet also determines the force acting on the droplet, which directly affects the stability and deformation of the levitated droplet.

#### 3.1.3. Simulation of Fluid Field

**Figure 4:**
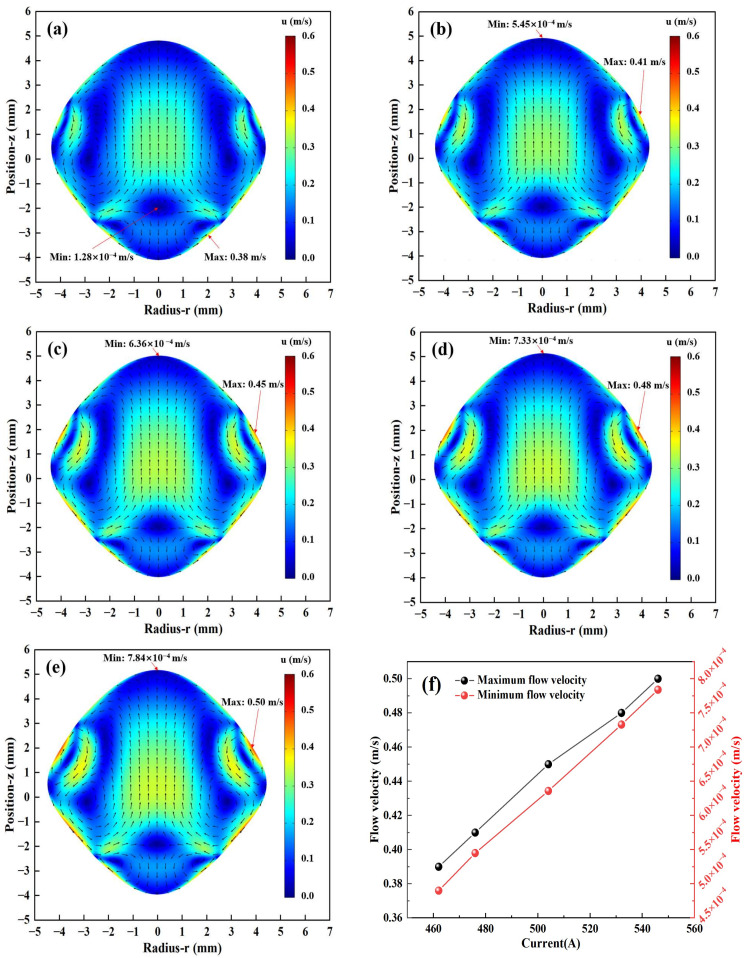
Simulation results of the fluid field at different currents: (**a**) 462 A; (**b**) 476 A; (**c**) 504 A; (**d**) 532 A; (**e**) 546 A; (**f**) Maximum and minimum flow velocity.

The effects of different currents on the fluid field inside the molten droplet of Ti-48Al-2Cr alloy are shown in Figure 4. The arrows in Figure 4 indicate the velocity field vectors, so based on the distribution of the velocity field vectors, it can be observed that the regions of higher flux are mainly located on the left, right, and center sides, as well as at the location of the vortices. Based on Equation (13), under the combined effect of Lorentz force and gravity, the fluid in the center region of the droplet flows upward, reaches the top, and then flows downward, two larger vortices are formed in the middle near the two sides, and two smaller vortices are formed in the bottom region. When the current is 462 A, four vortices can be observed, as shown in Figure 4a; and when the current increases from 476 A to 546 A, the left and right sides are increased by two small vortices, and the number of vortices is increased to six, as shown in Figure 4b–e. As shown in Figure 4f, the maximum and minimum flow velocities increase linearly with the current from 0.39 m/s to 0.48 m/s. In addition to the effect of the Lorentz force action, since the viscosity is inversely proportional to the temperature, the shear stress decreases with the temperature increase, which also promotes the increase in the flow velocity.

#### 3.1.4. Simulation of Thermal Field

**Figure 5:**
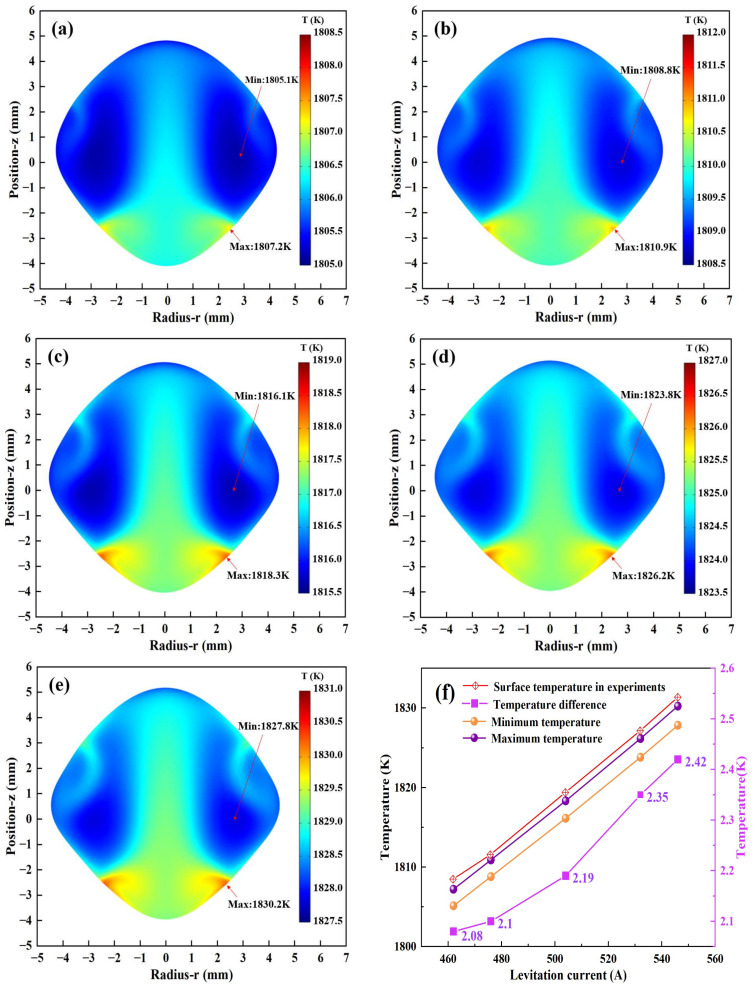
Simulation results of the temperature field at different currents: (**a**) 462 A; (**b**) 476 A; (**c**) 504 A; (**d**) 532 A; (**e**) 546 A; (**f**) temperatures.

Figure 5a–e show the numerical simulation results of the temperature distribution of the molten droplet. Based on the calculations in Equations (8) and (11), it can be seen that the bottom of the droplet near the heating coil obtains the maximum joule heat and the highest temperature, while the area near the two sides in the middle has the lowest temperature. The heat transfer inside the droplet is mainly convection heat transfer. It can be seen from Figure 5f that with the increase in exciting current, the temperature inside the droplet keeps increasing, from 1807 K to 1830 K. This is because the eddy current induced inside the droplet continues to increase, which makes the heating effect of electromagnetic induction more obvious. In addition, the effect of electromagnetic stirring makes the internal temperature distribution of the molten droplet more uniform, and the temperature difference is only about 2 K to 2.4 K. Comparing the surface temperatures of the molten droplets measured during the experiment with the maximum temperatures calculated by the simulation, we can see that the values are very close and the differences between them are less than 2 K, which proves the accuracy of the simulation.

### 3.2. Simulation of the Oscillation Process of Molten Droplets

**Figure 6:**
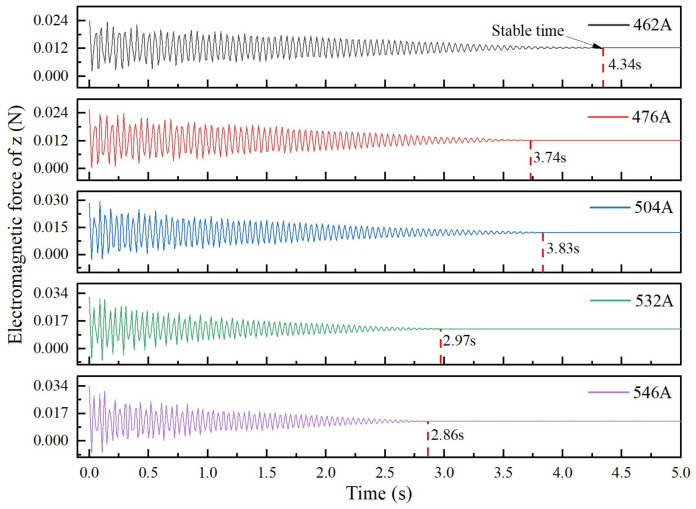
Variation curves of electromagnetic force on the molten droplets at different currents.

In the initial stage of levitation melting, there is a transient oscillation process that is affected by a variety of factors, such as the initial position, coil current, number of turns of the coil, and material properties [25,26]. The main influence on the oscillation process is the magnitude of the current when the initial position, coil structure, and current frequency are known. The droplet is mainly subjected to the Lorentz force and gravity, and the direction of the combined force is parallel to the *z*-axis. During the oscillation process, the Lorentz force on the droplet also changes due to the change in the levitation position. The oscillatory behavior of the electromagnetically levitated droplets can be approximated by the spring model theory. The relation between the displacement of the molten droplet near the equilibrium position and the combined force is consistent with the relation between the spring force applied to the suspension load $F_{s}$ and the spring deformation $x_{s}$ described in Hooke’s law, that is $F_{s}=-kx_{s}$, where $x_{s}$ is the spring displacement and *k* is the elastic coefficient. Figure 6 shows the curve of the electromagnetic force on the molten droplet at different currents, and it can be seen that the electromagnetic force in the *z*-axis direction gradually decreases with time until the stable trend, which is known as a damped oscillation. When the current is 462 A, the Lorentz force on the molten droplet stabilizes after 4.34 s, which is the longest time, and when the current is further increased to 546 A, the corresponding stabilization time decreases to 2.86 s. It can be concluded that the molten droplets perform the damped oscillatory motion in the electromagnetic levitation system, and with the increase in the current, the electromagnetic force on the molten droplets increases, the oscillation damping of the molten droplets gradually increases, and its stabilization time tends to decrease.

### 3.3. Simulation of the Deformation Process of Molten Droplets

**Figure 7:**
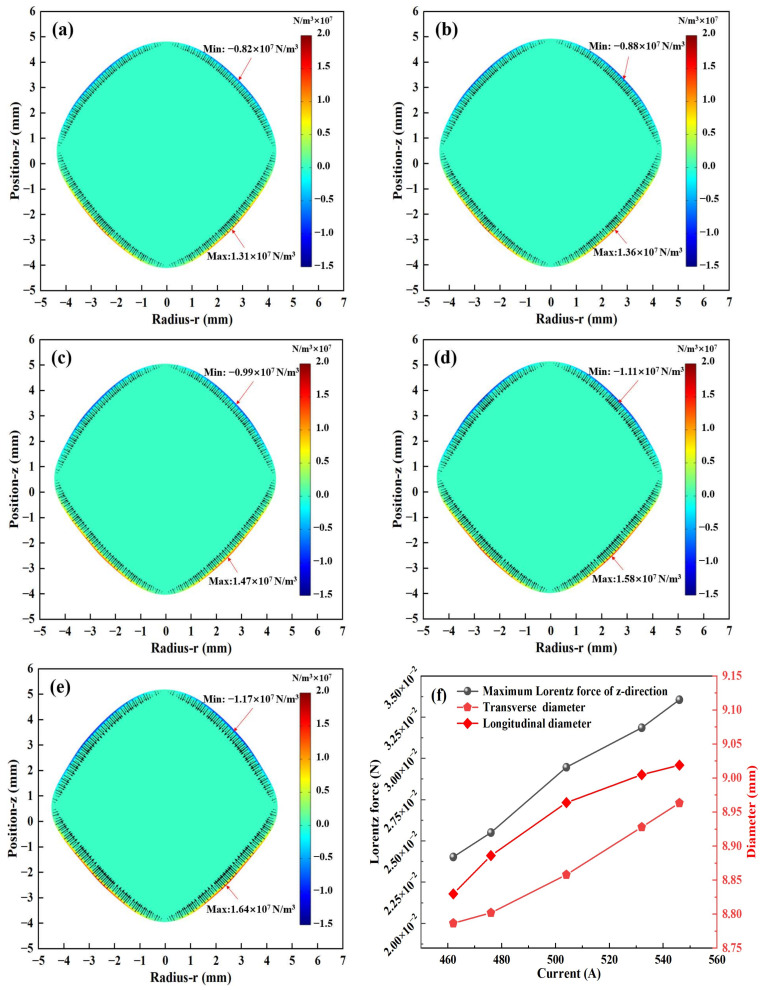
Lorentz force within the molten droplets at different currents: (**a**) 462 A; (**b**) 476 A; (**c**) 504 A; (**d**) 532 A; (**e**) 546 A; (**f**) maximum Lorentz force and the diameter of the molten droplets in different directions.

The distributions of the Lorentz force at different currents obtained by numerical simulation are shown in Figure 7a–e. As the current increases, the maximum Lorentz force increases from 1.27 × 10^7^ N/m^3^ to 1.58 × 10^7^ N/m^3^, and the Lorentz force on the droplet is mainly distributed on its surface. On the contrary, the Lorentz force inside the droplet tends to be close to 0 N/m^3^ due to the skinning effect of the high-frequency current induced. The Lorentz force on the upper and lower surfaces of the droplet is directed toward the inside of the droplet, and the arrangement of the coil structure makes the Lorentz force on the lower surface of the droplet significantly higher than that on the upper surface, and the maximum value occurs on the two sides near the lower part of the coil. Figure 7f shows the maximum Lorentz force corresponding to different levitation currents and the variation in the diameter in both the transverse and longitudinal directions. The transverse and longitudinal diameters of the droplets increase, but the change in the transverse diameter tends to flatten, and the shape eventually remains “near rhombus”.

**Figure 8:**
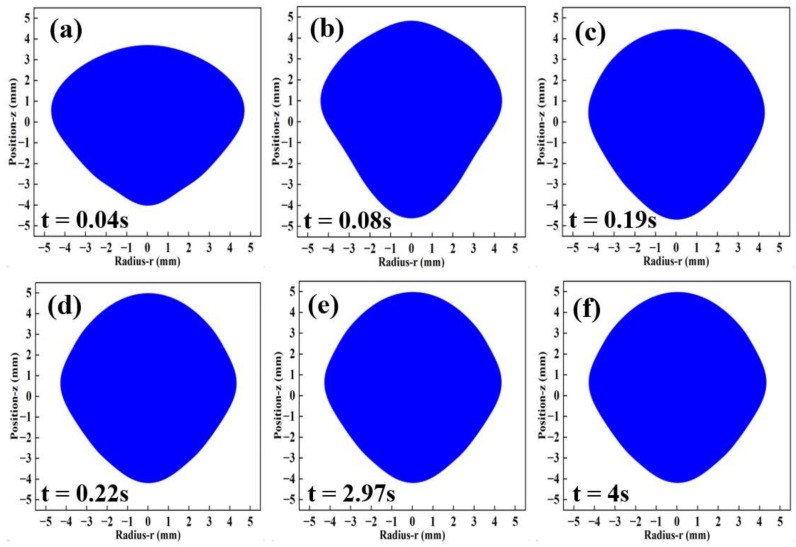
Deformation of the molten droplet in the interval from 0 s to 4 s at a current of 532 A: (**a**) 0.04 s; (**b**) 0.08 s; (**c**) 0.19 s; (**d**) 0.22 s; (**e**) 2.97 s; (**f**) 4 s.

To deeply analyze the deformation behavior during the oscillation of the molten droplet, the deformation process of the molten droplet was simulated from 0 s to 4 s at a current of 532 A, and the results are shown in Figure 8. From the previous analysis, it can be seen that the Lorentz force pushes the surface of the molten droplet inward and upward, and the component of the Lorentz force in the *z*-axis direction is opposite to gravity at the equilibrium position. At the initial stage of oscillation and deformation, the molten droplet starts from the initial position, the up and down oscillation amplitude is large, and the deformation is severe, as shown in Figure 8a–d. With the increase in time, the degree of oscillation and deformation is gradually weakened, and the molten droplet tends to be stable in shape at 2.97 s, showing a “near rhombus”, as shown in Figure 8e. Then, the shape at 4 s is unchanged compared with that at 2.97 s, as shown in Figure 8f.

**Figure 9:**
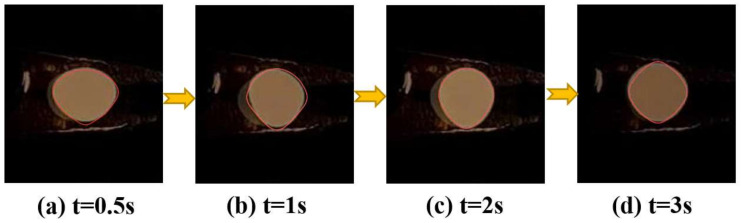
Deformation of the Ti-48Al-2Cr alloy molten droplet at different stages in the EML process (at the current of 532 A): (**a**) 0.5 s; (**b**) 1 s; (**c**) 2 s; (**d**) 3s.

As shown in Figure 9, the color photographs of the molten droplet of Ti-48Al-2Cr alloy in the process of EML (at the current of 532 A) were taken using a video camera. Figure 9a–c show the sample melting in the oscillating deformation stage. By comparison, it can be seen that the deformation is almost consistent with the simulation process in Figure 8, as shown by the red contour curves in each image. After about 3 s to 4 s, the oscillation and deformation tend to stabilize, and the shape of the molten droplet remains “near rhombus”, as shown in Figure 9d.

## 4. Conclusions

In this paper, numerical simulations of multiphysics field coupling and the oscillatory deformation of Ti-48Al-2Cr alloy under different current conditions during EML melting were investigated by the FEM and ALE methods. Finally, the comparative validation was carried out by EML experiments, and the following conclusions were obtained:

(1) The maximum velocity zones of the electromagnetically levitated droplets are located on the left and right sides near the droplet surface. Then, the number of vortices also rises from four to six with the current increase. The maximum temperature is observed at the bottom of the molten droplet, while the temperature near the center is relatively lower. The application of electromagnetic stirring results in a more uniform temperature distribution within the molten droplet, with a temperature difference of approximately 2 K.
(2) The titanium–aluminum alloy molten droplet is damped and oscillates from its initial position due to the combined effects of gravity and the Lorentz force. The amplitude of the oscillation increases with the current, but when the current increases from 462 A to 546 A, the levitation time to reach a steady state decreases gradually, from 4.34 s to 2.86 s.
(3) The integrated force field exerts a linear influence on the longitudinal diameter of the titanium–aluminum alloy molten droplet, which increases with the current. In contrast, the transverse diameter undergoes a gradual flattening. At the onset of oscillation, the molten droplet exhibits greater deformation, before reaching a stabilization period where its shape remains unchanged and assumes a “near-rhombus” configuration.

## Figures and Tables

**Table 1:** EML parameters of numerical simulation model [22,23].

Physical Properties of Titanium–Aluminum Alloy	
Melting point [K]	1773
Conductivity [S/m]	1.3 × 107
Relative permeability	5
Density [Kg/m3]	(−0.0005 × T + 4.2254) × 1000
Dynamic viscosity [Pa·s]	(0.06071 − (6.89 × 10−5) × T + (2.695 × 10−8) × T2 − (3.583 × 10−12) × T3

## Data Availability

Data are contained within the article.
